# A Genome-Wide Association Study and Complex Network Identify Four Core Hub Genes in Bipolar Disorder

**DOI:** 10.3390/ijms18122763

**Published:** 2017-12-19

**Authors:** Zengyan Xie, Xianyan Yang, Xiaoya Deng, Mingyue Ma, Kunxian Shu

**Affiliations:** Institute of Bioinformatics, Chongqing University of Posts and Telecommunications, Chongqing 400065, China; xiezy@cqupt.edu.cn (Z.X.); yxianyan@outlook.com (X.Y.); deng_xiaoya@outlook.com (X.D.); mamingyue@cqupt.edu.cn (M.M.)

**Keywords:** bipolar disorder, GWAS, functional enrichment analysis, network analysis

## Abstract

Bipolar disorder is a common and severe mental illness with unsolved pathophysiology. A genome-wide association study (GWAS) has been used to find a number of risk genes, but it is difficult for a GWAS to find genes indirectly associated with a disease. To find core hub genes, we introduce a network analysis after the GWAS was conducted. Six thousand four hundred fifty eight single nucleotide polymorphisms (SNPs) with *p* < 0.01 were sifted out from Wellcome Trust Case Control Consortium (WTCCC) dataset and mapped to 2045 genes, which are then compared with the protein–protein network. One hundred twelve genes with a degree >17 were chosen as hub genes from which five significant modules and four core hub genes (*FBXL13*, *WDFY2*, *bFGF*, and *MTHFD1L*) were found. These core hub genes have not been reported to be directly associated with BD but may function by interacting with genes directly related to BD. Our method engenders new thoughts on finding genes indirectly associated with, but important for, complex diseases.

## 1. Introduction

Bipolar disorder (BD) is a common and severe mental disorder characterized by alternative episodes of mania/hypomania and depression [1]. It affects 1–5% of the world’s population [2,3,4]. Genetic studies have shown that bipolar disorder is a complex genetic disease that involves the interaction of multiple genes and the environment. Genetic factors can account for up to 60–85% of the risk [5,6,7,8]. The strong genetic basis of BD inspires plenty of research focused on finding candidate genes or single nucleotide polymorphisms (SNPs) associated with this disease.

Over the past few decades, traditional family-based linkage analysis and population-based case–control association analysis have been common means of identifying bipolar disorder susceptibility genes. With the advent of the third-generation polymorphism genetic marker SNPs, genome-wide association studies (GWASs) have also been applied to large-scale scanning of new BD susceptibility gene loci and a number of genes, such as *CACAN1C* [9,10], *ANK3* [10,11], *SYNE1* [12], *CSMD1* [12], *ITIH1* [11], *KIT* [11], and *DGKH* [13], have been found.

GWASs have proven to be useful in finding susceptibility genes of diseases. However, when used alone, it is difficult to determine genes that have relatively high GWAS *p*-values but may play a role through interaction with the genes directly associated with BD. The complexity of the disease makes it even more difficult to elucidate its molecular mechanism. Therefore, although the previous study has found a lot of genetic factors with significant effects on BD, its molecular mechanism remains unresolved. In this case, a comprehensive analysis focusing on gene interactions and biological functions will provide valuable information to explore the pathogenesis of BD. It has been found that the distribution of genetic marker loci on chromosomes and the interaction between SNPs are one of the major genetic basis for complex diseases [14]. The gene network is often used to reveal complex relationships among genes.

Considering that complex mental phenotypes may be affected by many genes with small or mild effects rather than one or two genes with a major impact [15], a comprehensive analysis of the underlying genes in the pathway or network framework may provide more insights into its molecular mechanism. It will be more efficient to understand the role of genes in complex diseases using network study. Some methods have been developed in this area, but the problem is far from being solved. There is scarce known molecular interaction mechanism and systematic gene network analysis for BD. Construction of a gene interaction network can be used to explore the synergistic effect of multiple genes on BD.

In this study, we performed a GWAS to obtain BD-related genes and confirmed their function by functional enrichment analysis. To further explore the association between these genes and BD, a network was constructed using a human protein–protein interaction database, and the BD-risk genes identified in the GWAS were mapped onto the network to find core hub genes. This will provide more insight into the molecular mechanisms of BD by determining the core hub genes of the network.

## 2. Results

### 2.1. GWAS Results

**Figure 1:**
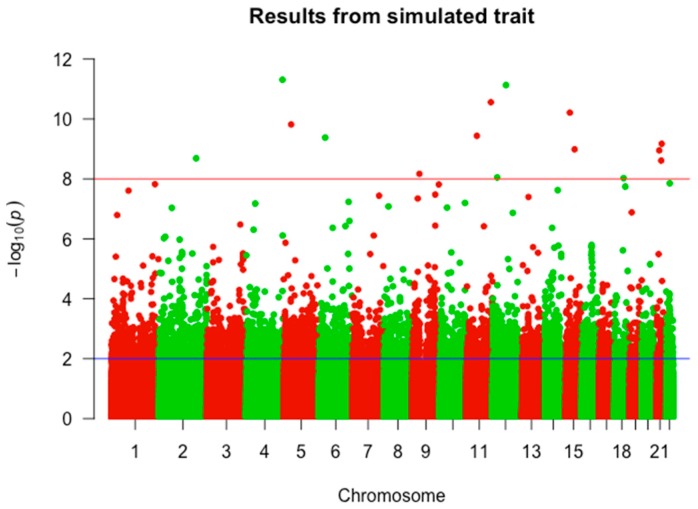
Results of the genome wide association study (GWAS). The horizontal axis represents 22 chromosomes and the vertical axis represents the negative logarithm with base 10 of GWAS *p*-value for each SNP. Red line: canonical 5 × 10^−8^ cutoff. Blue line: 0.01 cutoff used in this study.

A total of 482,247 SNPs located on 22 chromosomes of 1868 BD cases and 2938 controls satisfies the quality control. The number of SNPs decreases to 354,282 after the Hardy–Weinberg equilibrium test. Finally, a total of 6458 SNPs is qualified in the GWAS where *p* < 0.01 and used for further analysis. The result is shown in Figure 1.

### 2.2. Gene Functional Analysis

A total of 2045 risk genes was obtained after mapping the 6458 SNPs onto human genes. These genes were then classified into three Gene Ontology (GO) sections: cellular components, molecular functions, and biological processes. The first 10 GO items (*p* < 0.01) are shown in Table 1, Table 2 and Table 3. Genes with transferase and kinase function dominate in molecular functions. In cellular components, most gene products are located in the nervous system. This coordinates with the biological process result in which most genes are involved in nervous system development.

### 2.3. Overlapped Genes in Different Mental Illnesses

**Figure 2:**
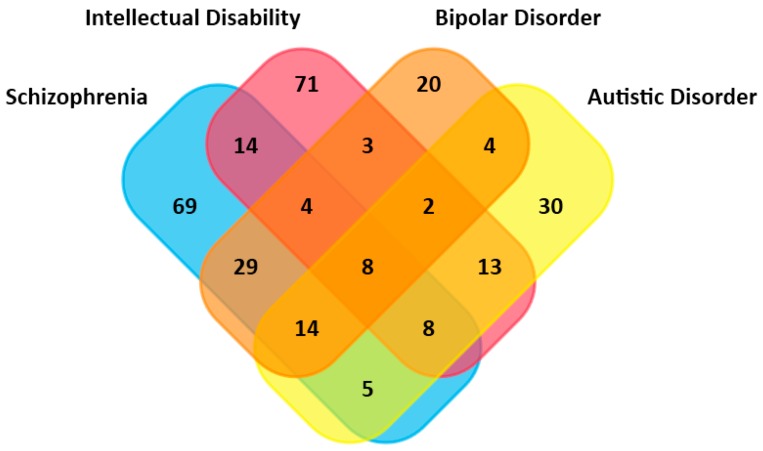
Overlapped genes associated with four mental illnesses.

We enriched these candidate genes in BD and other three mental illnesses: schizophrenia, intellectual disability, and autistic disorder. In the total 2045 risk genes, the numbers of genes associated with schizophrenia, intellectual disability, autism, and bipolar disorder are 151, 123, 84, and 84, respectively. A Venn map of the overlap genes of these four diseases shows that, out of the 84 genes associated with bipolar disorder, 55 genes are in common with schizophrenia, 17 with intellectual disorder, and 28 with autism (Figure 2).

### 2.4. Protein Interaction Network

**Figure 3:**
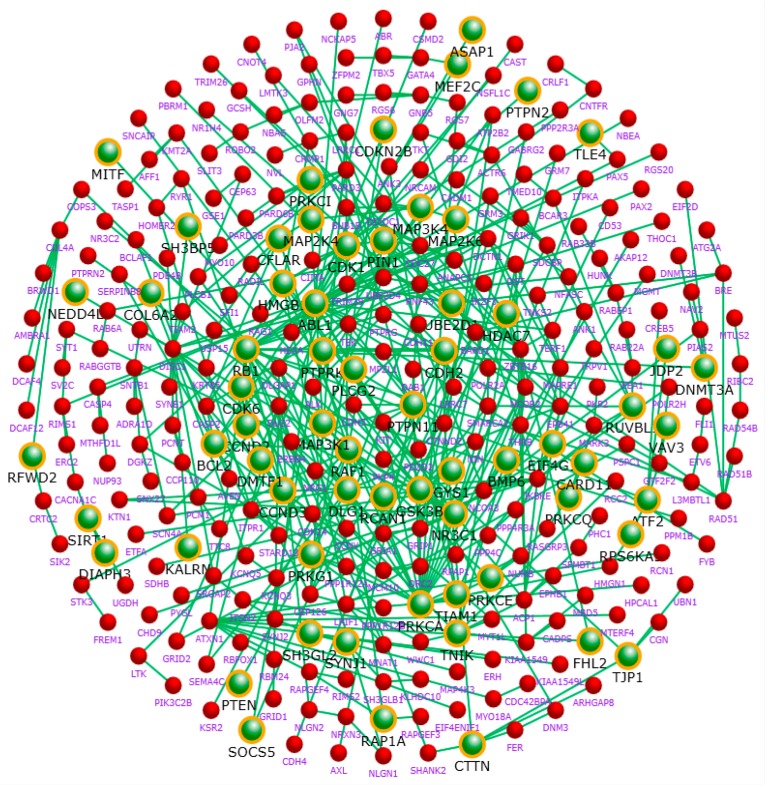
BD risk gene interaction network. Only the nodes with a degree ≥4 are shown. Green balls are BD risk genes identified in the GWAS with *p* < 0.01.

The 2045 genes from the GWAS result are mapped onto the protein–protein interaction network constructed using data from the STRING database (Figure 3).

**Figure 4:**
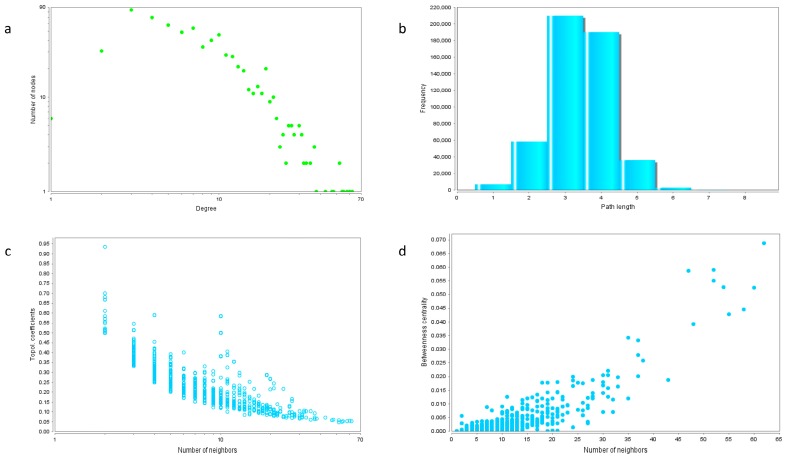
The topology properties of the network. (**a**) The distribution of number of nodes with different degrees. (**b**) Frequency distribution of shortest paths. (**c**) The relationship between topological coefficients and the number of node neighbors. (**d**) The relationship between betweenness and the number of node neighbors.

There are 1083 nodes in the network. The average node degree of the network is 7.555. The clustering coefficient is 0.232, and the characteristic path length is 3.393. The properties of the network are further analyzed and the results are shown in Figure 4. The connectivity of the network exhibits characteristic power distribution. Figure 4b shows that the shortest path with the highest frequency among the candidate genes of BD is between 3 and 4, indicating that the network is not a stochastic network but a complex network with characteristics of biological molecular network. The number of neighbors shared by the network nodes has a significant inverse relationship with its topological coefficients (Figure 4c), but shows a positive correlation with the node’s identity (Figure 4d).

### 2.5. Hub Genes of the Network

One hundred twelve gene nodes with a degree >17 is chosen as hub genes from the network for further analysis (Table 4 and Table A1).

Of these 112 hub genes, 45 were reported associated with BD in previous studies. Another 24 were reported associated with other mental illnesses. Gene nodes with higher degrees have a higher ratio of genes being reported associated with BD. Only five genes with a degree >23 (51 genes) are not reported associated with BD or other mental illnesses, while 24 with a degree ≤23 (61 genes) are not found reported directly associated to any mental illnesses. Obviously, risk genes with more degrees have a closer connection to BD than those with fewer degrees.

### 2.6. Significant Modules of the Network and Core Hub Genes

**Figure 5:**
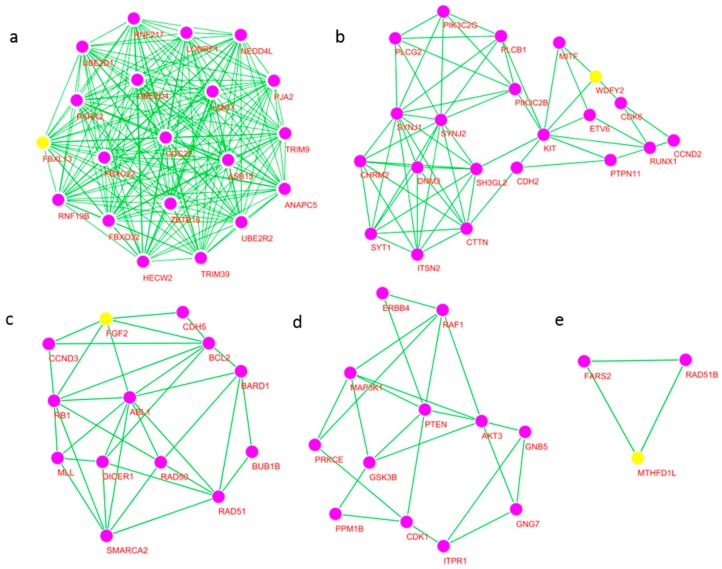
Gene clusters identified with Cytoscape. Yellow nodes are core hub genes. No core hub gene is found in Cluster 4 (**d**). (**a**) Cluster 1; (**b**) Cluster 2; (**c**) Cluster 3; (**d**) Cluster 4; (**e**) Cluster 5.

Five significant gene modules are found in the network containing 112 hub genes with Cytoscape. Four core hub genes are found in these modules: *FBXL13*, *WDFY2*, *bFGF* (*FGF2*), and *MTHFD1L*. No core hub gene is found for one module (Cluster 4) (Table 5, Figure 5).

## 3. Discussion

### 3.1. Most BD Risk Gene Products Are Located in the Nervous System

Our gene functional analysis of 2045 BD risk genes shows that most of their products are located in the nervous system, such as synapse and postsynapse. The result of GO biological process analysis shows most genes are involved in nervous system development. These two results verify each other and are consistent with previous studies [104]. BD risk genes may affect patients in two aspects: short-term and permanent. Environmental or internal factors may cause ectopic expression of some of the risk genes, which in turn cause episodes of BD. Some genes may work in the development of the nervous system and have a permanent effect on patients. This may explain why 60% patients will relapse into depression or mania within two years after treatment [105].

### 3.2. Intense Overlappings of Genes Associated with BD and Other Mental Disorders

Many symptoms and signs overlap between different mental disorders and patients often present with features of more than one disorder [106]. This may be caused by underlying genetic reasons. We compared BD risk genes with those of three other mental disorders and found intense overlaps. Similar results were also reported in other studies [28,52,86,89,106,107].

Schizophrenia and BD share the most associated genes. Previous work also found a significant correlation between a BP polygenic risk score and the clinical dimension of mania in schizophrenia patients [86]. *PRKG1* was reported to be significantly associated with schizophrenia. In this study, we also find it is a hub gene in the network of BD risk genes. This gene encodes a cGMP-dependent protein kinase which acts as key mediator of the nitric oxide (NO)/cGMP signaling pathway. Another gene, *SMARCA2*, was also found to play a role in the pathophysiology of schizophrenia [27]. Its product is a transcription activator and involved in neuron differentiation. Many other risk genes are also found involved in signal transduction and nervous system development. This suggests that these two diseases may share some common underlying pathways.

### 3.3. Core Hub Genes Give New Insights of BD

We combined protein–protein network and genome wide association analysis in this study and found four core hub genes. Although genes with higher degrees are more frequently reported to be associated with BD, two core hub genes (*WDFY2* and *FBXL13*) have relatively low degrees (20 and 19, respectively).

*bFGF* has not been reported to be associated with BD before, but is usually used for treatment of neurodegenerative diseases such as Alzheimer’s disease [42]. It plays an essential role in regulation of cell proliferation, differentiation, and migration. *bFGF* is found as a core hub gene implies the abnormal nervous development of BD patients.

There is no obvious evidence for another core hub gene, *MTHFD1L*, to be associated with BD, but it is thought to have an important effect on the pathophysiology of depression through rumination, and maybe via this cognitive intermediate phenotype on other mental and physical disorders [38].

*WDFY2* is not directly associated with BD, but its product interacts with *AKT1* [108], which has been found involved in BD and schizophrenia [109]. This result suggests that the pathophysiology of BD is even more complicated than we thought. Some genes may play a role through its interaction with genes directly associated with BD.

*FBXL13* functions in the maturation of human dendritic cells [68] which are key regulators in the immune system and show mild aberrancies in bipolar disorder that can be fully restored to even activation after in vivo lithium treatment [67].

Interestingly, all the four core hub genes are not directly associated with BD. Although the role of these genes in the pathophysiology of BD requires further investigation, our method inspires new initiatives to find those genes that are important for BD but overlooked by studies using GWAS alone.

### 3.4. Effectiveness of GWAS Followed by Gene Network Analysis

GWAS is a successful tool for identifying human disease-associated genes. However, results of different studies often vary due to sampling even when a strict significant *p*-value of 5 × 10^−8^ is used [110]. In this study, a loose *p*-value threshold of 0.01 was used for the GWAS, and a gene network analysis was then used to find BD-associated genes in the GWAS result. Many resulted genes with high network degrees but relatively high GWAS *p*-values are reported to be associated with BD and/or other mental illnesses (Table 4), which suggests that the combination of the two methods is efficient in finding disease-related genes. It is necessary to use a loose *p*-value threshold in the first step to provide enough input genes for the following network analysis. A second screening using network degrees can help to make the final result more reliable.

Sklar et al. conducted a combined GWAS with 7481 BD cases and 9250 controls and identified *CACNA1C* and a miRNA located in the first intron of *ODZ4* as BD-associated genes [87]. The calcium channel subunit coding gene *CACNA1C* has also been found to be associated with BD in previous studies [9,52,86] and is confirmed with a relatively high degree (30) in our results. However, the miRNA is not detected in this study, probably due to our relatively smaller sample size.

## 4. Materials and Methods

### 4.1. Bipolar Disorder Datasets

The dataset is from a study published by Wellcome Trust Case Control Consortium (WTCCC), which conducted a genome-wide scan of all SNPs of 17,000 British Caucasian loci by human SNPs genotyping chips. This dataset includes 14,000 disease samples from seven common complex diseases: bipolar disorder, bipolar depression, Crohn’s disease, hypertension, rheumatoid arthritis, type 1 diabetes, type 2 diabetes, and 3000 healthy control samples, which has been completed by more than 50 research teams [111]. The dataset is downloaded from WTCCC website [112]. This study uses the BD part of the dataset. Human SNP annotation data and human reference sequence data are downloaded from NCBI (https://www.ncbi.nlm.nih.gov/), which contain 336,843,011 SNPs on 24 human chromosomes and the start and end of genes in which they are located [113].

### 4.2. Screening of Risk SNPs

SNP sites that do not meet one of the following criteria are excluded for quality control: Hardy–Weinberg equilibrium test (Bonferroni corrected *p* < 5 × 10^−7^), missingness >5%, minor allele frequency <5%, and odds ratio *R*^2^ > 0.8. Risk SNPs are screened under *p* < 0.01. Quality control and risk gene screening are finished with Plink software [114].

### 4.3. Mapping Significant Risk SNPs to Genes

Risk SNPs are mapped onto human genes by comparing them with transcription start sites and stop sites. An SNP will be mapped onto its nearest gene within 5 kb if it is not located within any gene. SNPs located outside of the 5 kb of genes are removed.

### 4.4. Gene Function and Disease Enrichment Analysis

FunRich [115] software is used to carry out gene enrichment analysis with *p* < 0.01. Results are reversely ordered by FDR-values, and only the first 10 results are listed in each GO section. ToppGene [116] is used to enrich genes in four different mental illnesses.

### 4.5. Protein Network Analysis

STRING database [117] is used to find a protein–protein relationship and FunRich is then used to map BD risk genes to the protein–protein network. Those protein (gene) nodes with degree >17 are sifted out as hub genes, which are further analyzed with the MCODE plugin [118] of Cytoscape [119] to find out network clusters (modules) and core hub genes. The node gene with the highest MCODE node score in a cluster is designated as its core hub gene, which is crucial for the cluster.

The topological properties of a gene cluster include [120,121] the following: (1) degree, the number of genes directly connected to a gene, (2) the cluster coefficient (CC), the coincidence of the common regulatory genes between two adjacent genes, defined as

(1) $$\mathrm{CC}=\frac{2n_{i}}{k_{i}\left(k_{i}-1\right)}$$

where $n_{i}$ represents the number of edges of the $k_{i}$ neighbors that connect to node i—the mean of the clustering coefficients of all nodes is designated as the clustering coefficient of the network—(3) the shortest path, the path with the least edges between two nodes, and (4) betweenness (B(*v*)), the sum of the ratios of number of shortest paths connecting to a node to that of all shortest paths in a network

(2) $$B\left(\nu \right)=\underset{s\neq \nu ,s\neq t,\nu \neq t\in \nu }{\sum }\frac{\delta_{st}\left(\nu \right)}{\delta_{st}}$$

where $\delta_{st}$ is total number of shortest paths from node *s* to *t*, and $\delta_{st}\left(\nu \right)$ is the number of those paths that pass through *v*.

## Figures and Tables

**Table 1:** Molecular functions (GO).

Name	FDR	Gene Count
transferase activity, transferring phosphorus-containing groups	5.67 × 10^−5^	118
kinase activity	5.67 × 10^−5^	103
phosphotransferase activity, alcohol group as acceptor	5.67 × 10^−5^	96
protein serine/threonine kinase activity	1.03 × 10^−^^4^	63
protein kinase activity	1.90 × 10^−^^4^	81
GTPase regulator activity	2.61 × 10^−^^4^	47
signal transducer activity, downstream of receptor	2.61 × 10^−^^4^	33
GTPase activator activity	4.57 × 10^−^^4^	43
adenyl ribonucleotide binding	7.16 × 10^−^^4^	154

**Table 2:** Cellular components (GO).

Name	FDR	Gene Count
synapse	5.23 × 10^−14^	131
postsynapse	1.68 × 10^−13^	83
synapse part	7.30 × 10^−13^	110
synaptic membrane	1.89 × 10^−12^	62
cell junction	9.04 × 10^−10^	149
postsynaptic embrane	1.91 × 10^−9^	47
neuron part	2.94 × 10^−9^	178
excitatory synapse	7.86 × 10^−9^	49
plasma membrane region	8.87 × 10^−9^	130
neuron projection	8.89 × 10^−9^	144

**Table 3:** Biological processes (GO).

Name	FDR	Gene Count
neurogenesis	1.28 × 10^−7^	186
cell morphogenesis	1.28 × 10^−^^7^	159
generation of neurons	1.28 × 10^−^^7^	176
regulation of nervous system development	1.28 × 10^−^^7^	116
neuron differentiation	3.55 × 10^−^^7^	162
neuron development	3.55 × 10^−^^7^	136
cell projection morphogenesis	3.61 × 10^−^^7^	116
cellular component morphogenesis	4.18 × 10^−^^7^	164
cell projection organization	4.63 × 10^−^^7^	163
neuron projection morphogenesis	6.25 × 10^−^^7^	88

**Table 4:** The gene nodes with a network degree >17.

Hub Gene	Degree	Hub Gene	Degree	Hub Gene	Degree
*CDK1*	62	*PNPLA6* [16]	27	*FARS2*	20
*PTEN* [17,18]	61	*SYNJ2*	27	*FBXO22*	20
*BCL2* [19]	60	*UBE2R2* [20]	27	*FLT3*	20
*POLR2A* [21]	55	*CACNA1D* [22,23,24]	26	*GATA4* [25] *	20
*SMARCA2* [26,27]	55	*CDK6* [28]	26	*ITSN2* [29]	20
*GSK3B* [30,31,32,33]	54	*CHRM2* [34]	26	*KIF18A*	20
*ABL1* [35,36,37]	53	*MTHFD1L* [38] **	26	*LONRF1*	20
*PRKCA* [39,40]	50	*GRIA1* [41]	25	*NCOA3*	20
*bFGF* [42] **	48	*POLR2H*	25	*PCNT* [43]	20
*RB1* [44] *	45	*TJP1* [45] *	25	*PJA2*	20
*KIT* [11]	40	*MAPRE1* [46] *	24	*SYT1* [47]	20
*RAD51* *	38	*RUNX1* [48]	24	*TRIM39*	20
*SIRT1* [49,50,51]	38	*UBE2D4*	24	*WDFY2* [52] **	20
*UBE2D1* [53,54]	37	*EHHADH*	23	*AK4*	19
*DLG1* [55,56]	36	*IQCB1*	23	*ASB15*	19
*CDC27* [57]	35	*PPM1B* [58]	23	*ATF2* [29]	19
*NEDD4L* [59]	35	*PPP4C* [60] *	23	*BUB1B*	19
*PRKG1* [61] *	35	*RAD50*	23	*DHX15*	19
*RAP1A*	34	*SH3GL2*	23	*DNM3* [62] *	19
*CDH2* [63] *	33	*DCTN1*	22	*ETV6*	19
*GNB5* [64] *	33	*ERBB4* [65,66]	22	*FBXL13* [67,68] **	19
*MAPK6* [69]	33	*FBXO32*	22	*HECW2*	19
*GNG7* [70]	32	*ITPR1* [71] *	22	*MEF2C* [72] *	19
*PTPN11* [73] *	32	*MLL* [74]	22	*NR3C1* [75]	19
*ZBTB16* [76]	32	*NCOR2* [77]	22	*BDE4D*	19
*ADCY2* [78]	31	*PRKCE* [79]	22	*RNF19B*	19
*DICER1* [80,81]	31	*RAD51B*	22	*RNF217*	19
*SYNJ1* [82,83,84,85]	31	*ACTN4*	21	*RXFP2*	19
*CACNA1C* [9,52,86,87]	30	*CCND2* [88,89]	21	*RYR1*	19
*CTTN*	30	*CDH5*	21	*THBS2*	19
*DLG2* [90,91]	30	*CUL4A* [92] *	21	*AKT3* [93] *	18
*MAP3K1* [94] *	30	*EFCAB13*	21	*BARD1*	18
*RIT2* [95]	30	*LMO7*	21	*CTNNA2* [11,96]	18
*ANAPC5* [28]	28	*MITF*	21	*HDAC7*	18
*PLCB1* [97,98,99]	28	*TRIM9* [100]	21	*ITGAV*	18
*RAF1* [101] *	28	*CCND3*	20	*PARD3* [102] *	18
*PARK2* [61] *	27	*EPHB1* [103] *	20	*PCSK2*	18
*PLCG2* [13,39]	27				

* associated with other mental illness ** core hub genes.

**Table 5:** Significant risk gene modules.

Cluster	Score	Nodes	Edges	Node IDs
1	20	20	190	*ASB15*, *HECW2*, *UBE2D1*, *NEDD4L*, *ANAPC5*, *PJA2* *TRIM39*, *UBE2R2*, *UBE2D4*, *CDC27*, *TRIM9*, *ZBTB16* *LONRF1*, *PARK2*, *FBXL13* *, *FBXO22*, *RNF19B*, *LMO7* *RNF217*, *FBXO32*
2	6.1	21	61	*SYNJ1*, *KIT*, *PIK3C2G*, *PTPN11*, *PIK3C2B*, *SYNJ2* *RUNX1*, *ITSN2*, *PLCB1*, *CDH2*, *DNM3*, *SYT1*, *CTTN WDFY2* *, *CHRM2*, *CCND2*, *MITF*, *PLCG2*, *CDK6*, *ETV6*, *SH3GL2*
3	5.5	13	33	*MLL*, *bFGF(FGF2)* *, *BUB1B*, *BARD1*, *RB1*, *DICER1*, *RAD50*, *RAD51*, *BCL2*, *CDH5*, *SMARCA2*, *ABL1*, *CCND3*
4	4.182	12	23	*AKT3*, *PTEN*, *ITPR1*, *PRKCE*, *GNB5*, *CDK1* *ERBB4*, *GNG7*, *RAF1*, *GSK3B*, *PPM1B*, *MAP3K1*
5	3	3	3	*FARS2*, *RAD51B*, *MTHFD1L* *

* core hub genes.

## Abbreviations

BD	bipolar disorder
GWAS	genome-wide association study
SNP	single nucleotide polymorphism
WTCCC	the World Healthcare Case Control Association

## Appendix A

**Table A1:** Gene information of the nodes with a network degree greater than 17.

GENE	Degree	Ensembl	UniProtKB
*CDK1*	62	ENSG00000170312	P06493
*PTEN*	61	ENSG00000171862	P60484
*BCL2*	60	ENSG00000171791	P10415
*POLR2A*	55	ENSG00000181222	P24928
*SMARCA2*	55	ENSG00000080503	P5153
*GSK3B*	54	ENSG00000082701	P49841
*ABL1*	53	ENSG00000097007	P00519
*PRKCA*	50	ENSG00000154229	P17252
*FGF2*	48	ENSG00000138685	P09038
*RB1*	45	ENSG00000139687	P06400
*KIT*	40	ENSG00000157404	P10721
*RAD51*	38	ENSG00000051180	Q06609
*SIRT1*	38	ENSG00000096717	Q96EB6
*UBE2D1*	37	ENSG00000072401	P51668
*DLG1*	36	ENSG00000075711	Q12959
*CDC27*	35	ENSG00000004897	P30260
*NEDD4L*	35	ENSG00000049759	Q96PU5
*PRKG1*	35	ENSG00000185532	Q13976
*RAP1A*	34	ENSG00000116473	P62834
*CDH2*	33	ENSG00000170558	P19022
*GNB5*	33	ENSG00000069966	O14775
*MAPK6*	33	ENSG00000069956	Q16659
*GNG7*	32	ENSG00000176533	O60262
*PTPN11*	32	ENSG00000179295	Q06124
*ZBTB16*	32	ENSG00000109906	Q05516
*ADCY8*	31	ENSG00000155897	P40145
*DICER1*	31	ENSG00000100697	Q9UPY3
*SYNJ1*	31	ENSG00000159082	O43426
*CACNA1C*	30	ENSG00000151067	Q13936
*CTTN*	30	ENSG00000085733	Q14247
*DLG2*	30	ENSG00000150672	Q15700
*MAP3K1*	30	ENSG00000095015	Q13233
*RIT2*	30	ENSG00000152214	Q99578
*ANAPC5*	28	ENSG00000089053	Q9UJX4
*PLCB1*	28	ENSG00000182621	Q9NQ66
*RAF1*	28	ENSG00000132155	P04049
*PARK2*	27	ENSG00000185345	O60260
*PLCG2*	27	ENSG00000197943	P16885
*PNPLA6*	27	ENSG00000032444	Q8IY17
*SYNJ2*	27	ENSG00000078269	O15056
*UBE2R2*	27	ENSG00000107341	Q712K3
*CACNA1D*	26	ENSG00000157388	Q01668
*CDK6*	26	ENSG00000105810	Q00534
*CHRM2*	26	ENSG00000181072	P08172
*MTHFD1L*	26	ENSG00000120254	Q6UB35
*GRIA1*	25	ENSG00000155511	P42261
*POLR2H*	25	ENSG00000163882	P52434
*TJP1*	25	ENSG00000104067	Q07157
*MAPRE1*	24	ENSG00000101367	Q15691
*RUNX1*	24	ENSG00000159216	Q01196
*UBE2D4*	24	ENSG00000078967	Q9Y2X8
*EHHADH*	23	ENSG00000113790	Q08426
*IQCB1*	23	ENSG00000173226	Q15051
*PPM1B*	23	ENSG00000138032	O75688
*PPP4C*	23	ENSG00000149923	P60510
*RAD50*	23	ENSG00000113522	Q92878
*SH3GL2*	23	ENSG00000107295	Q99962
*DCTN1*	22	ENSG00000204843	Q14203
*ERBB4*	22	ENSG00000178568	Q15303
*FBXO32*	22	ENSG00000156804	Q969P5
*ITPR1*	22	ENSG00000150995	Q14643
*MLL*	22	ENSG00000118058	Q03164
*NCOR2*	22	ENSG00000196498	Q9Y618
*PRKCE*	22	ENSG00000171132	Q02156
*RAD51B*	22	ENSG00000182185	O15315
*ACTN4*	21	ENSG00000130402	O43707
*CCND2*	21	ENSG00000118971	P30279
*CDH5*	21	ENSG00000179776	P33151
*CUL4A*	21	ENSG00000139842	Q13619
*EFCAB13*	21	ENSG00000178852	Q8IY85
*LMO7*	21	ENSG00000136153	Q8WWI1
*MITF*	21	ENSG00000187098	O75030
*TRIM9*	21	ENSG00000100505	Q9C026
*CCND3*	20	ENSG00000112576	P30281
*EPHB1*	20	ENSG00000154928	P54762
*FARS2*	20	ENSG00000145982	O95363
*FBXO22*	20	ENSG00000167196	Q8NEZ5
*FLT3*	20	ENSG00000122025	P36888
*GATA4*	20	ENSG00000136574	P43694
*ITSN2*	20	ENSG00000198399	Q9NZM3
*KIF18A*	20	ENSG00000121621	Q8NI77
*LONRF1*	20	ENSG00000154359	Q17RB8
*NCOA3*	20	ENSG00000124151	Q9Y6Q9
*PCNT*	20	ENSG00000160299	O95613
*PJA2*	20	ENSG00000198961	O43164
*SYT1*	20	ENSG00000067715	P21579
*TRIM39*	20	ENSG00000204599	Q9HCM9
*WDFY2*	20	ENSG00000139668	Q96P53
*AK4*	19	ENSG00000162433	P27144
*ASB15*	19	ENSG00000146809	Q8WXK1
*ATF2*	19	ENSG00000115966	P15336
*BUB1B*	19	ENSG00000156970	O60566
*DHX15*	19	ENSG00000109606	O43143
*DNM3*	19	ENSG00000197959	Q9UQ16
*ETV6*	19	ENSG00000139083	P41212
*FBXL13*	19	ENSG00000161040	Q8NEE6
*HECW2*	19	ENSG00000138411	Q9P2P5
*MEF2C*	19	ENSG00000081189	Q06413
*NR3C1*	19	ENSG00000113580	P04150
*PDE4D*	19	ENSG00000113448	Q08499
*RNF19B*	19	ENSG00000116514	Q6ZMZ0
*RNF217*	19	ENSG00000146373	Q8TC41
*RXFP2*	19	ENSG00000133105	Q8WXD0
*RYR1*	19	ENSG00000196218	P21817
*THBS2*	19	ENSG00000186340	P35442
*AKT3*	18	ENSG00000117020	Q9Y243
*BARD1*	18	ENSG00000138376	Q99728
*CTNNA2*	18	ENSG00000066032	P26232
*HDAC7*	18	ENSG00000061273	Q8WUI4
*ITGAV*	18	ENSG00000138448	P06756
*PARD3*	18	ENSG00000148498	Q8TEW0
*PCSK2*	18	ENSG00000125851	P16519
*PIK3C2B*	18	ENSG00000133056	O00750
*PIK3C2G*	18	ENSG00000139144	O75747
*UBQLN1*	18	ENSG00000135018	Q9UMX0
